# Dental disease in rabbits under UK primary veterinary care: Clinical management and associated welfare impacts

**DOI:** 10.1002/vetr.5326

**Published:** 2025-04-03

**Authors:** Maria A. Jackson, Dan G. O'Neill, Joanna Hedley, Dave C. Brodbelt, Charlotte C. Burn

**Affiliations:** ^1^ Pathobiology and Population Sciences The Royal Veterinary College Hatfield UK; ^2^ Beaumont Sainsbury Animal Hospital The Royal Veterinary College London UK

**Keywords:** animal welfare, *Oryctolagus cuniculus*, pain, teeth, VetCompass

## Abstract

**Background:**

Dental disease is a painful and highly prevalent condition in companion rabbits. However, the dental disease management techniques currently employed by UK primary‐care veterinarians and potential associated clinical welfare implications are scarcely described.

**Methods:**

Anonymised clinical records from primary‐care practices participating in the VetCompass programme in 2019 were manually reviewed to identify cases of dental disease in rabbits. Clinical welfare implications were assessed through retrospective analysis of clinical signs, diagnostics and treatment information.

**Results:**

A total of 2219 rabbit dental disease cases were recorded in 2019. The most frequently recorded clinical signs were reduced food intake (25.1%) and reduced faecal output (10.9%). Diagnostic dental radiography was performed in 2.2% of cases. Tooth trimming (including using burs, rasps and nail clippers) was conducted on 34.0% of cases; 6.1% of rabbits undergoing cheek teeth trimming had the procedure performed while conscious. Dietary modification was recommended for 21.5% of cases. Dental disease was the primary reason for death or a contributory factor in 51.2% of the cases that died.

**Limitations:**

Accurate dental disease diagnosis relies on detailed veterinary examination and confidence in diagnosing rabbit dental disease, which may vary.

**Conclusion:**

Dental disease is a major welfare concern for rabbits, as indicated by the high frequency of detrimental clinical signs, the potentially suboptimal treatment methods used in some cases and its frequency as a full or contributory cause of death. Greater owner and veterinarian awareness of dental disease signs and further veterinary education on appropriate diagnostic and treatment methods could improve the welfare of affected rabbits.

## INTRODUCTION

Dental disease is common in domestic rabbits, with a reported annual prevalence of 15.4% and annual incidence risk of 8.2% in companion rabbits under UK primary veterinary care in 2019.^1^ Despite the high frequency of dental disease in rabbits and literature reporting that many veterinarians have low confidence in their pain assessment, diagnosis and treatment of rabbits,^2^, ^3^, ^4^ the clinical techniques commonly employed by veterinarians in the management of rabbit dental disease are scarcely described. Valuable insights into some aspects of welfare in affected rabbits may be gained from a retrospective analysis of clinical records that describe clinical signs, veterinary diagnostic methods used and treatments employed.

Dental disease has the potential to cause suffering in rabbits, as indicated by the range of clinical signs previously suggested to occur with the disease and related co‐morbidities (Table 1). Previous studies in Thailand, Chile and the United States have provided some evidence that companion rabbits with dental disease frequently experience reduced food intake, gastrointestinal hypomotility and ocular discharge.^5^, ^6^, ^7^ However, those studies were limited by relatively small sample sizes, and the type of veterinary practice (primary care or referral) that rabbits were examined at is unclear. Assessment of the presenting signs and clinical examination findings in rabbits with dental disease may elucidate the extent to which rabbits may experience pain, hunger and weakness from having damaged and dysfunctional teeth. It could also indicate the type and severity of dental disease and potential co‐morbidities. Utilising records of rabbits attending primary‐care veterinary practices can help identify any common clinical signs for rabbits experiencing dental disease that veterinarians and owners can look out for.

**TABLE 1:** Clinical signs commonly reported in rabbits with dental disease

Body system affected	Specific clinical sign
Oral	Facial abscesses with no signs of trauma
	Horizontal enamel ridges on incisors (ribbing)
	Hypersalivation (drooling, slobbers)
	Oral ulceration and/or bleeding
	Palpable swellings on the mandible or maxilla
	Skull asymmetry
	Uneven wear and spurs on cheek teeth
	Visibly overgrown incisors
	Inability to close mouth due to overgrown teeth
	Dysphagia or choking on unchewed food
	Altered jaw movement patterns
Gastrointestinal	Secondary gastrointestinal stasis due to pain and/or nutritional inadequacies
	Weight loss (or weight gain)
Dermatological	Alopecia around the mouth and/or eyes
	Caecotroph accumulation around the perineum
Ocular	Exophthalmos
	Recurrent or chronic epiphora or dacryocystitis
Respiratory	Purulent nasal discharge
Behavioural	Reduced food intake (anorexia, hyporexia)
	Reluctance to eat hay and other hard foods
	Change in drinking habits (e.g., may prefer bowls to bottles)
	Dropping uneaten food
	Aggression from pain
	Lethargy from pain
	Pawing at mouth
	Reduced bruxism
	Reduced grooming (or excessive grooming)

*Note*: Adapted from Crossley^8^; Capello^9^; Verstraete and Osofsky^10^; Harcourt‐Brown^11^; Lennox^12^; Harcourt‐Brown,^13^; and Böhmer^14^

Although recommended diagnostic and treatment methods for rabbits with dental disease have been described in the literature, to date, the diagnostic and treatment methods actually employed in companion rabbit dental disease cases under UK primary veterinary care have not been systematically described. Documentation of currently used techniques would be beneficial to identify possible opportunities for improvement. In addition to using visual oral examination with an otoscope or similar device to assist dental disease diagnosis,^8^, ^12^, ^15^, ^16^, ^17^ recommendations have been made for using radiography or other imaging modalities such as computed tomography (CT), under sedation or general anaesthesia, to more accurately detect subgingival dental changes.^8^, ^9^, ^10^, ^12^, ^13^, ^14^, ^15^, ^16^, ^17^, ^18^, ^19^, ^20^ To illustrate, in a small Finnish study of 67 rabbits with acquired dental disease, 36 rabbits were diagnosed with the disease during oral examinations, whereas 64 showed evidence of dental disease in lateral skull radiography, indicating the diagnostic sensitivity and value of radiography.^21^

For all rabbits, providing an appropriate, high‐fibre diet is crucial for preventing or slowing the progression of acquired dental disease,^5^, ^6^, ^8^, ^13^, ^14^, ^22^, ^23^, ^24^, ^25^, ^26^ and may be the only treatment recommended for uncomplicated tooth elongation.^16^ Other treatments to correct tooth malocclusion include tooth height reduction (trimming), although the progressive nature of acquired dental disease means tooth trimming may be necessary at regular intervals throughout the affected rabbit's life.^8^, ^9^, ^10^, ^12^, ^16^, ^23^, ^24^ Repeated trimming can increase the potential for rabbit stress during restraint,^27^ which is potentially exacerbated when carried out by an unfamiliar clinician in unfamiliar surroundings. It is widely advised that trimming of cheek teeth should be performed under sedation or general anaesthesia to reduce unnecessary stress and reduce the risk of potentially welfare‐compromising iatrogenic injury.^10^, ^12^, ^16^, ^23^, ^24^, ^28^, ^29^ Trimming incisors may be performed with dental burs, but the use of nail clippers is specifically discouraged because it carries a high risk of shattering the teeth, causing severe pain and secondary infection.^8^, ^12^, ^13^, ^16^, ^23^, ^30^, ^31^, ^32^ Surgical extraction of teeth, particularly incisors, may be considered in certain cases where repeated tooth trimming is undesirable,^12^, ^16^, ^23^ though cheek teeth extraction is less commonly recommended due to difficulty and a high risk of complications.^8^, ^9^, ^10^, ^12^, ^16^, ^24^

Sedation and general anaesthesia during diagnostic and treatment procedures bring some welfare costs and health risks, such as postanaesthetic gastrointestinal complications^33^ and a higher risk of death in rabbits than in anaesthetised cats or dogs.^34^ However, sedation and anaesthesia also offer some welfare benefits, including reduced stress and pain during diagnostic procedures^35^, ^36^ and better visualisation during oral examination,^8^, ^9^, ^15^, ^16^ likely providing a greater opportunity for more accurate diagnosis and targeted veterinary treatment.

Euthanasia may be indicated for rabbits suffering from dental disease when their reduction in quality of life becomes severe,^8^, ^14^, ^23^, ^37^ perhaps from dental pain and resulting emaciation if a rabbit loses their willingness or physical ability to eat, or as a result of co‐morbidities. Dental disease can co‐occur with or even promote a range of co‐morbidities, including myiasis and gastrointestinal stasis,^12^, ^16^, ^37^ which are likely to cause discomfort or pain^38^, ^39^, ^40^, ^41^ and are frequent causes of death in companion rabbits.^42^ Assessment of the frequency of death in rabbits with dental disease and whether dental disease was implicated in their deaths can provide information on the severity of the disease.

Through descriptive analysis of retrospective clinical data from a companion rabbit population, this study aimed to describe the presenting clinical signs of rabbits with dental disease, diagnostic and treatment methods used by UK primary‐care veterinarians, and mortality associated with the disease. Additionally, the frequency of case referrals for advanced clinical management was recorded to gauge primary‐care veterinarians' general confidence in dental disease treatment. Data on whether dietary modification was recommended for affected rabbits were also extracted to identify veterinary awareness and application of this important aspect of dental disease prevention. The welfare implications of dental disease itself, and to what extent commonly used veterinary therapies affect welfare, were also inferred through the assessment of these clinical benchmarking results.

## METHODS

The current study was an extension of previous work described by Jackson et al.,^1^ using the same case population. The study population comprised all 161,979 rabbits under primary veterinary care at clinics participating in the VetCompass programme in 2019.^43^ VetCompass collates de‐identified electronic health records (EHRs) for epidemiological research from over 30% of primary‐care practices across the UK. Data fields available for the current study included a unique identifying number, species, breed, date of birth, sex, neuter status and bodyweight, as well as free‐text clinical notes containing diagnostic terms and treatments, with relevant dates. Ethical approval was obtained from the Royal Veterinary College Ethics and Welfare committee (reference number: SR2018‐1652).

All study rabbits were initially screened to identify candidate dental disease cases. The clinical free‐text fields of EHRs were searched using optimised search terms with word fuzziness that allowed two‐letter insertion or deletion,^44^ including dent*, ‘cheek teeth’, burr* and extraction ∼1. A randomly selected subset of the resulting candidate clinical records was reviewed manually to evaluate for case inclusion.^45^ Randomisation used the RAND (transact‐SQL) function within SQL Server. A dental disease case was defined as any rabbit with evidence in the available clinical records of the presence of any dental abnormality at any point between 1 January and 31 December 2019. Rabbits diagnosed with dental disease before 2019 but without recorded evidence of ongoing dental disease during 2019 were not included as cases. Diagnostic decision making was at the discretion of the attending veterinarians.

For confirmed cases of dental disease, details of the clinical signs, diagnostic methods used and treatment information were manually extracted by completing data extraction questions for each dental disease case. Data extraction questions were piloted on a subset of 20 EHRs, and questions were refined to optimise the quantity of data extracted in the available timeframe. The affected teeth were recorded as incisors (including peg teeth), cheek teeth (molars and premolars) or unrecorded. Any clinical signs (owner reported or identified by the veterinarian during clinical examination) were recorded if they were documented in the EHR within 7 days following dental disease diagnosis, but may or may not have been directly related to the dental disease. A list of 34 clinical signs was created during the pilot phase, and the full list of clinical signs and associated definitions used in this study can be seen in Supporting Information S1.

Diagnostic methods documented in the EHR to have occurred within 7 days of the rabbit meeting the dental disease case definition were extracted. These included visual oral examination (with or without the recorded use of an otoscope), radiography, CT, magnetic resonance imaging (MRI) or postmortem examination. All intra‐oral dental treatment documented to occur specifically for the treatment of dental disease at any point up until 31 December 2019 was recorded. This included tooth trimming or tooth extraction, but not surgical treatment of the oral mucosa on its own without dental involvement. Tooth trimming was defined as any clipping, filing, rasping, burring or other technique used to reduce the height or change the shape of the occlusal surface of any teeth. The documented use of sedation and general anaesthesia was also recorded for the diagnostic visual oral examination, radiography, CT, MRI and tooth trimming events. Any documented recommendation of dietary modification in relation to the rabbit's dental disease made within 7 days of meeting the dental disease case definition was recorded. Data on referral for advanced clinical management for dental disease were also extracted, which included treatment within or outside the original practice at any point during the available clinical records (including after 31 December 2019); however, further diagnostics or treatments performed at a different veterinary practice following the referral were not commonly available and thus not extracted.

Rabbits confirmed as dental disease cases and that had died at any point in the available records (including after 31 December 2019) had the contribution of dental disease to their cause of death recorded as follows:

- *Primary cause*: The dental disease was the dominant reason why the rabbit died or was euthanased; for example, the rabbit was euthanased due to poor prognosis from overgrown teeth, or died during anaesthesia to provide dental treatment.
- *Contributory cause*: Dental disease was recorded as one of a series of contributory factors leading to the rabbit's death or euthanasia; for example, the rabbit died with gastrointestinal stasis but was recorded to have overgrown cheek teeth concurrently.
- *Unrelated cause*: The rabbit died from an unrelated reason with no mention of dental disease as a contributory factor in their death or euthanasia; for example, the rabbit died following a fox attack.
- *Unrecorded cause*: No cause of death was recorded.

Euthanasia was not recorded separately from other mechanisms of death. Data were processed in Microsoft Excel (2022), and descriptive analyses were conducted using IBM SPSS Statistics (V28.0.1.1).

## RESULTS

### General clinical information

From the study population of 161,979 rabbits, 44,089 candidate cases were identified based on the search terms applied to the EHR. A random sample of 3935 EHRs (8.9% of the candidate cases) was reviewed in detail, and 2219 cases met the dental disease case definition. Of these 2219 confirmed dental disease cases, 1034 (46.6%) were pre‐existing to 2019 and 1185 (53.4%) were incident cases in 2019 (Table 2). Cheek teeth alone were reported as affected in 1726 rabbits (77.8%), only incisors affected in 198 (8.9%), both cheek teeth and incisors affected in 256 (11.5%) and an unrecorded tooth type(s) in the remaining 39 rabbits (1.8%).

**TABLE 2:** Clinical information relating to confirmed dental disease in 2219 rabbits under primary veterinary care in the UK in 2019 at practices registered with the VetCompass programme

Clinical variable	Category	Number of cases	Percentage (%)
Incident or pre‐existing cases	Incident in 2019	1185	53.4
	Pre‐existing	1034	46.6
Tooth type affected	Incisors only	198	8.9
	Cheek teeth only	1726	77.8
	Incisors and cheek teeth	256	11.5
	Unrecorded	39	1.8
At least one clinical sign recorded	Yes	1096	49.4
	No	1123	50.6
Any diagnostic technique performed	Yes	2175	98.0
	No	44	2.0
Any intra‐oral dental treatment performed	Yes	787	35.5
	No	1432	64.5
Dietary modification recommended	Yes	476	21.5
	No	1743	78.5
Referral for advanced clinical management recorded	Yes	83	3.7
	No	2136	96.3
Rabbit death recorded	Yes	547	24.7
	No	1672	75.3

### Clinical signs of dental disease

**FIGURE 1:**
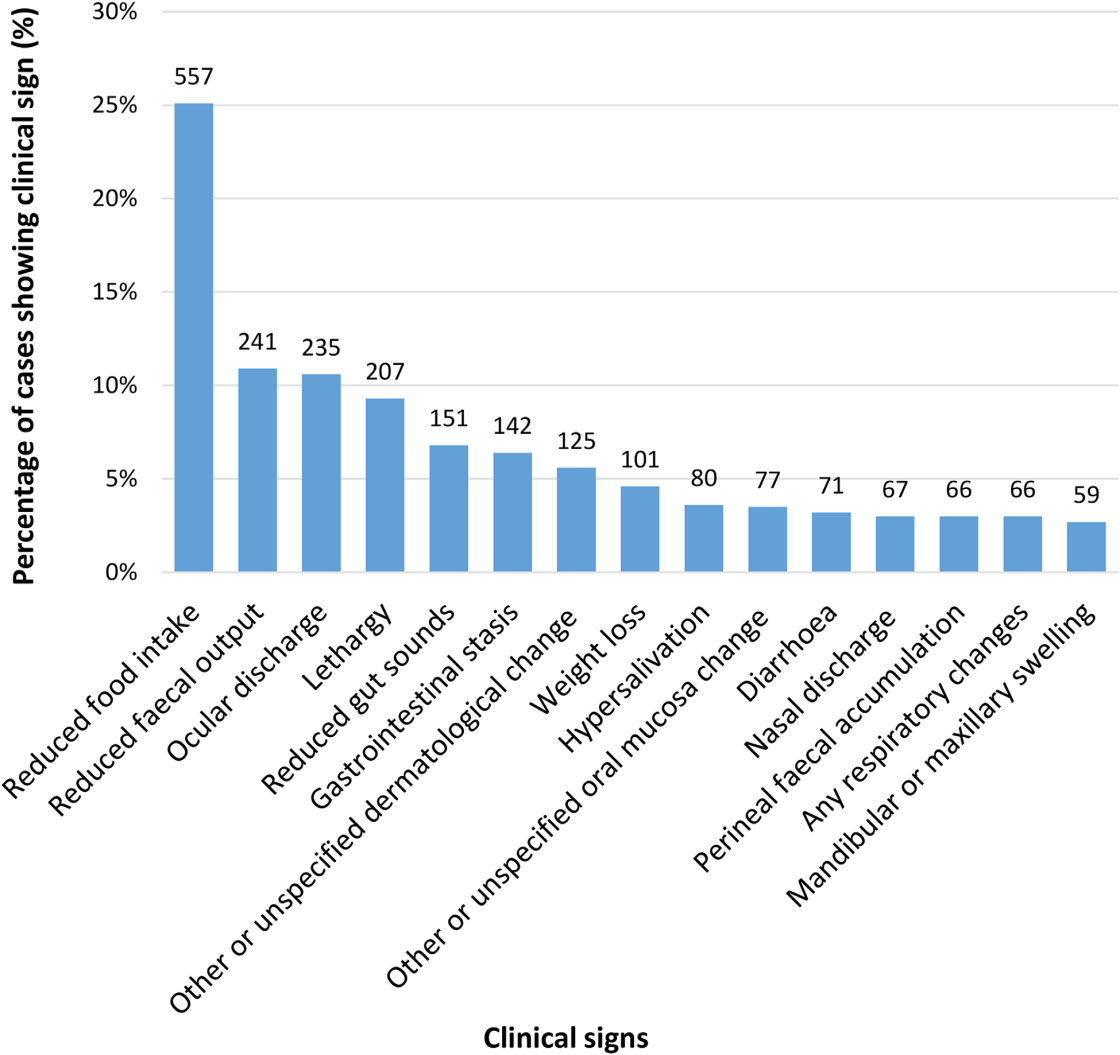
The most frequently recorded clinical signs in rabbit dental disease cases (*n* = 2219) under primary veterinary care in the UK in 2019. ‘Other or unspecified dermatological change’ included any mention of skin issues other than perineal faecal accumulation, myiasis or under/overgrooming; this may have included pruritus, alopecia and/or mite infestation. ‘Other or unspecified oral mucosa change’ included any mention of oral mucosal damage other than oral bleeding, ulceration or abscessation; this may have included gingival hyperplasia or tongue lacerations. Full clinical sign definitions are given in Supporting Information S1

Overall, 1096 dental disease cases (49.4%) had at least one clinical sign recorded within 7 days of dental disease diagnosis. Reduced food intake was reported in 557 rabbits, comprising 25.1% of all 2219 cases and 50.8% of the 1096 rabbits with at least one clinical sign recorded. Reduced faecal output was the next most common clinical sign, reported in 10.9% of all cases and 22.0% of those with at least one recorded clinical sign, followed by ocular discharge, reported in 10.6% of all cases and 21.4% of those with at least one recorded clinical sign. The 15 most frequently recorded clinical signs are shown in Figure 1.

### Diagnostic methods

**FIGURE 2:**
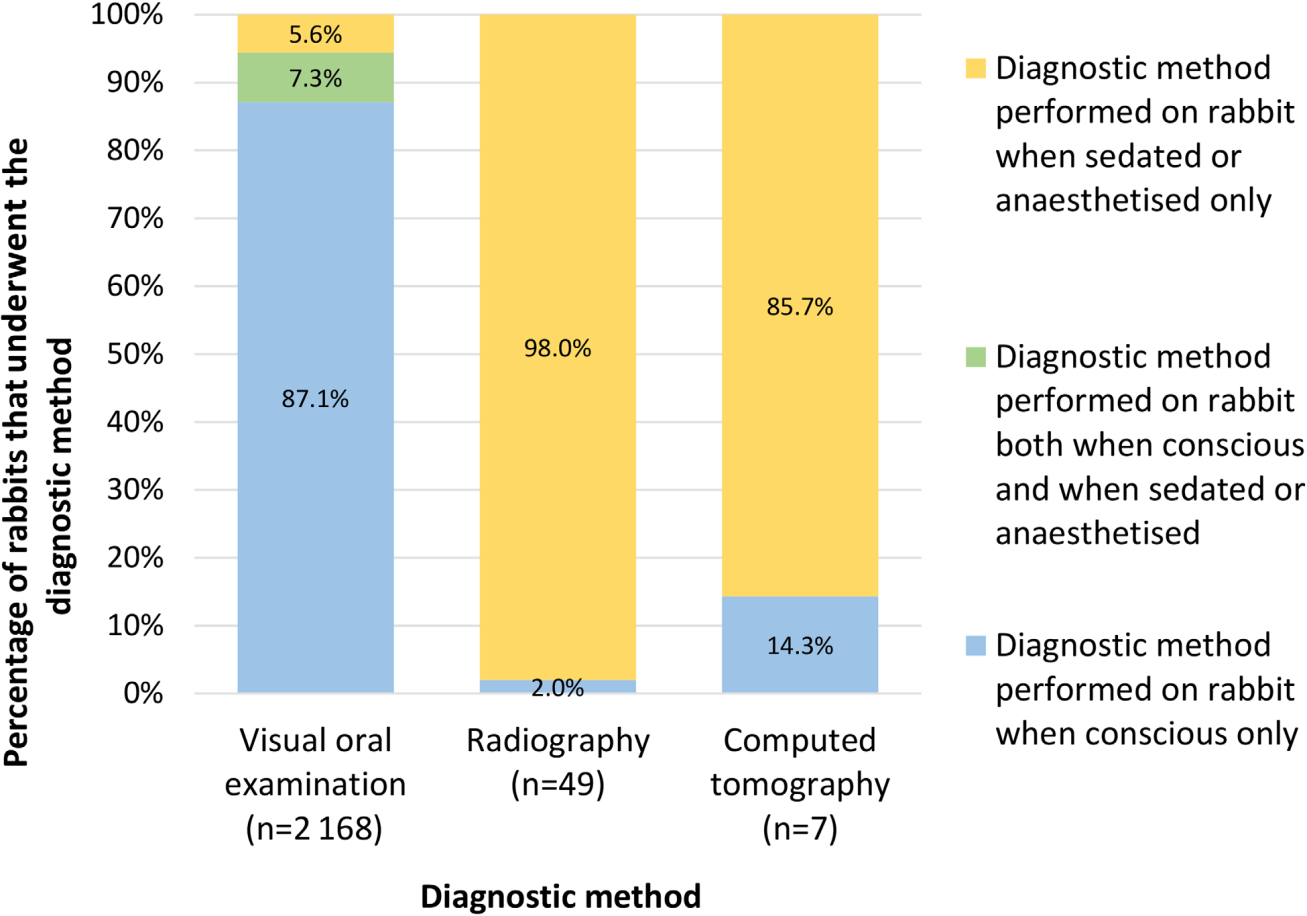
Percentages of rabbits undergoing visual oral examination, radiography and computed tomography while conscious or under sedation or general anaesthesia, from the 2175 cases of dental disease that had at least one diagnostic method recorded. Individual rabbits with dental disease may have undergone multiple types of diagnostic methods

At least one diagnostic method was used to assist in confirmation of dental disease within 7 days of diagnosis in 2175 (98.0%) cases. Visual oral examination was recorded as performed in 2168 of these 2175 (99.7%) cases. Of the 2175 cases with diagnostic methods recorded, radiography was used in 49 (2.2%) and CT in seven (0.3%). One rabbit underwent postmortem examination, and none were recorded as having an MRI. The proportions of rabbits undergoing the three most common diagnostic methods while conscious or under sedation or general anaesthesia are displayed in Figure 2.

### Treatments and veterinary recommendations

**FIGURE 3:**
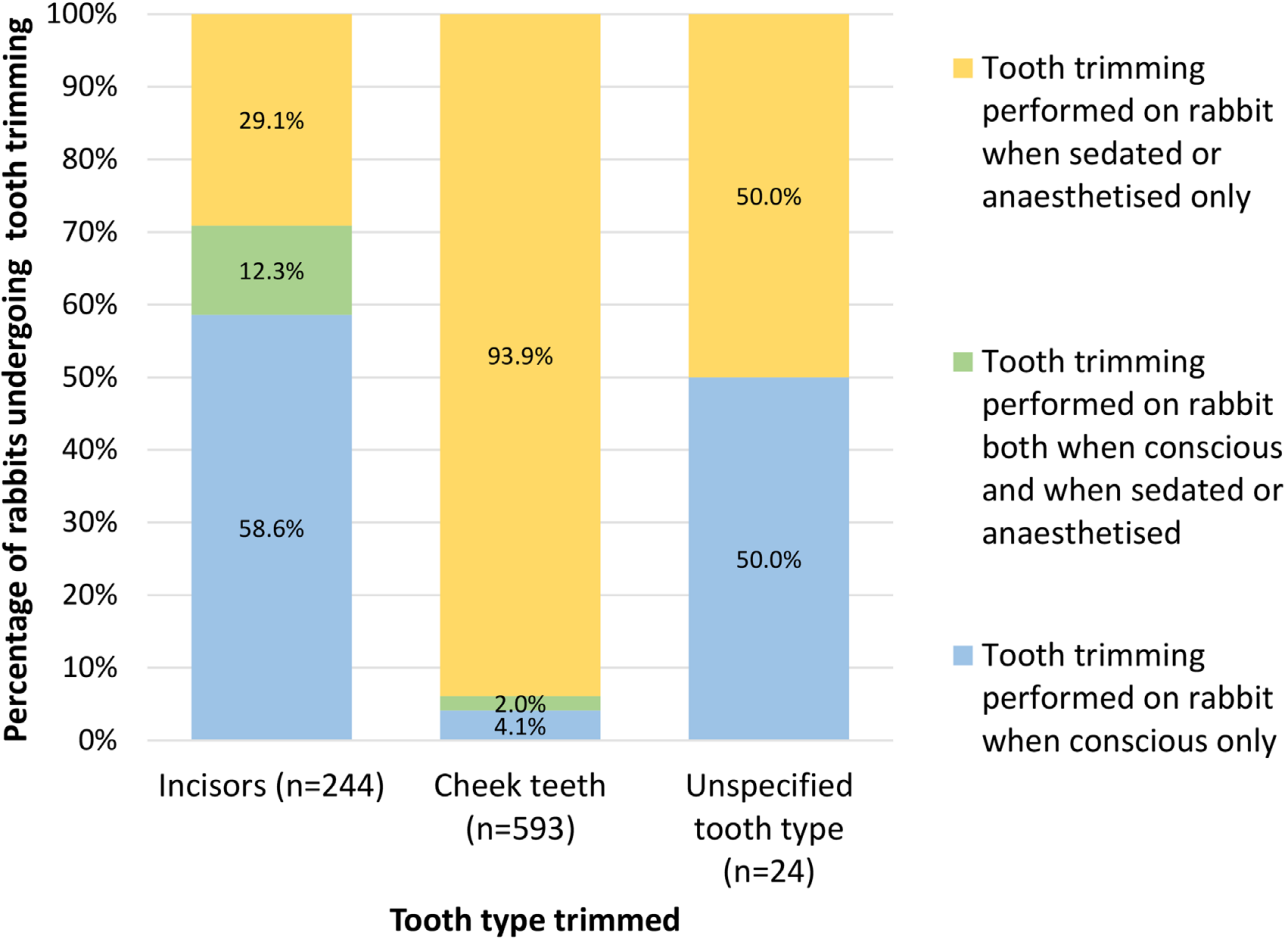
Percentages of rabbits undergoing tooth trimming events while conscious or under sedation or general anaesthesia, from the 754 cases of dental disease that underwent tooth trimming. The number of rabbits that underwent trimming of a specified tooth type is shown in brackets next to that tooth type. Some rabbits with dental disease underwent tooth trimming on both incisors and cheek teeth (*n* = 102)

Intra‐oral dental treatment at any point until 31 December 2019 was recorded in 787 (35.5%) cases. Tooth trimming was recorded in 754 (34.0%) cases, with 244 (32.4%) rabbits undergoing incisor trimming, 593 (78.6%) rabbits undergoing cheek teeth trimming and 24 (3.2%) rabbits undergoing tooth trimming where the tooth type was unspecified; 102 (13.5%) rabbits had both incisors and cheek teeth trimmed. In some cases, nail clippers were reportedly used for tooth trimming, but the frequency of their use was not extracted separately from other trimming methods because the majority of EHRs did not specify the trimming method. The proportions of tooth trimming events that occurred with the rabbit conscious or under sedation or general anaesthesia are displayed in Figure 3, with 173 (70.9%) incisor and 36 (6.1%) cheek teeth trimming events performed on conscious rabbits.

A total of 126 rabbits (5.7% of the total dental disease cases) underwent tooth extraction at any point until 31 December 2019. These 126 tooth extraction cases included 86 (68.3%) incisor extraction events, 46 (36.5%) cheek teeth extraction events and three (2.4%) unspecified tooth type extraction events; nine (7.1%) rabbits underwent both incisor and cheek teeth extraction.

A recommendation for dietary modification in relation to dental disease within 7 days of diagnosis was recorded for 476 rabbits (21.5% of the total dental disease cases), and 83 (3.7%) rabbits were referred for advanced clinical management at any point in the available clinical record.

### Mortality

In all, 547 rabbits (24.7%) were recorded to have died at any point in the available EHR. Dental disease was the primary cause of death for 92 (16.8%) of these rabbits, and a contributory cause for a further 188 rabbits (34.4%). The cause of death was not related to dental disease for 158 rabbits (28.9%), and there was no recorded cause of death for the remaining 109 (19.9%).

## DISCUSSION

This study aimed to describe the clinical management and outcomes of companion rabbits with dental disease, based on real‐world veterinary clinical data from the UK, and then to explore these findings to offer an inference on the likely welfare impacts of both dental disease itself and commonly used veterinary clinical techniques. Based on a detailed review of anonymised clinical records from 2219 rabbits diagnosed with dental disease, the authors identified some findings of welfare concern, including a high frequency of detrimental clinical signs. Some opportunities for welfare improvement were also identified, such as the occurrence of conscious cheek tooth‐trimming events. The tooth types affected by dental disease, clinical signs, diagnostic and treatment methods, and mortality described here will be discussed in the context of their possible implications for rabbit welfare.

### Tooth types affected by dental disease

Of rabbits with reported dental disease, 77.8% had only cheek teeth affected, 11.5% also had dental disease affecting the incisors and a further 1.8% had unspecified tooth types affected. The health status of cheek teeth cannot easily be assessed without the use of an otoscope or similar intra‐oral viewing device owing to rabbits’ long narrow oral cavities,^8^ so without regular veterinary oral examinations, many owners may be unaware of dental abnormalities on their rabbit's cheek teeth, particularly as rabbits often hide signs of pain and illness.^12^, ^14^, ^46^, ^47^ Acquired dental disease in rabbits is a progressive disorder,^11^, ^22^, ^48^ and later dental changes are often irreversible,^8^ so without regular dental examinations and appropriate veterinary intervention, initially minor disorders of the cheek teeth are likely to progress. Rabbits with overgrown teeth will likely experience decreased welfare from the pain of tooth root apices pressing on the nerve supply to teeth when eating abrasive food such as hay,^22^, ^48^ and their consequent masticatory impairment may result in nutritional deficiencies and frustration at a reduced ability to graze.^49^, ^50^, ^51^, ^52^ Rabbits may not have been presented for veterinary care until advanced stages are reached where clinical signs of cheek teeth disease are noticeable, perhaps explaining the higher frequency of dental disease cases affecting cheek teeth than incisors in this study. Likewise, cases of dental disease affecting cheek teeth may have been higher than reported here due to the low use of radiography or CT for diagnosis.

The relatively high frequency of reported cheek teeth disease in the current study, in comparison to incisor disease, could also reflect misdiagnosis of normal cheek teeth sharp edges as abnormal spurring,^1^, ^11^, ^16^, ^22^, ^53^ thus inflating the number of recorded cases of dental disease affecting cheek teeth. Specific tooth pathology and abnormalities could not be assessed in the current study but do warrant further research. Either way, work is needed to encourage rabbit owners to present their animals for regular intra‐oral examinations to identify abnormal dental changes early. Owners should also monitor their rabbits for early evidence of dental disease, such as epiphora or reluctance to eat hay.^22^, ^48^

### Clinical signs of dental disease

The most common clinical signs recorded in rabbits within 7 days of dental disease diagnosis were reduced food intake (25.1%) and reduced faecal output (10.9%). Many of the 13 other most frequent clinical signs associated with dental disease in the current study were also related to gastrointestinal disorders. Normal gastrointestinal function in rabbits relies on the ingestion of large quantities of fibre to aid peristalsis, so difficulties in prehending and masticating hay, including reduced food intake, are likely to predispose rabbits to gastrointestinal hypomotility and stasis.^8^, ^41^, ^52^, ^54^, ^55^ Gastrointestinal stasis can be acutely painful for rabbits and, if left untreated, can lead to hepatic lipidosis and liver failure and, eventually, death.^40^, ^41^, ^52^, ^56^, ^57^, ^58^ In a previous retrospective study of UK companion rabbits under primary veterinary care, ileus (another term for gastrointestinal stasis) was reported as the cause of death for 4.3% of a sample of 370 rabbits, and anorexia was the cause of death for 4.9%.^42^ Optimal treatment for rabbits with dental disease should therefore focus on supporting gastrointestinal motility and providing analgesia to reduce pain, as rabbits experiencing dental pain from overgrown tooth roots may be reluctant to eat hay.^48^

In addition to frequent gastrointestinal signs, ocular discharge was also recorded in 10.6% of dental disease cases and lethargy in 9.3%. The type of ocular discharge was not distinguished in the current study, but epiphora (abnormal tear overflow onto the face) and dacryocystitis (inflammation of the lacrimal sac and/or nasolacrimal duct, usually involving bacterial infection) often typically follow nasolacrimal duct obstruction from elongated tooth roots.^59^, ^60^, ^61^, ^62^ Ocular discharge may predispose rabbits to discomfort from moisture‐associated dermatitis around the eyes, as well as self‐trauma from any irritation.^59^ Moreover, the lethargy recorded in 9.3% of dental disease cases could indicate pain^63^, ^64^ or weakness from insufficient food consumption in affected rabbits. Additional studies are warranted to assess rabbit chronic pain behaviours related to dental disease, as recent literature regarding rabbit pain signs is generally related to acute or postoperative pain,^65^, ^66^, ^67^, ^68^ and chronic pain is notoriously difficult to detect in prey species such as rabbits.^64^, ^69^, ^70^ Chronic pain can also be accompanied by depression‐like behaviour, as seen in people and laboratory rodents,^71^ and possibly fear or anxiety.^69^ Rabbit owners should therefore be advised to monitor for ocular discharge or subtle behavioural changes, including lethargy, as possible indicators of dental disease in order to initiate rapid treatment and reduction of pain and associated negative welfare states.

### Diagnostic methods

In the current study, diagnostic imaging was used very infrequently in the diagnosis of dental disease, with just 2.2% of diagnoses using radiography and 0.3% using CT. As previously mentioned, oral examination under sedation or general anaesthesia^8^, ^9^, ^10^, ^12^, ^14^, ^15^, ^16^, ^24^, ^36^ and radiography^8^, ^9^, ^10^, ^12^, ^13^, ^14^, ^15^, ^16^, ^17^, ^18^, ^20^, ^24^ are widely recommended for more accurate dental disease diagnosis and staging and to aid in the planning of appropriate treatment. The low frequency of diagnostic imaging used in this population may reflect financial challenges; just 23% of rabbits were estimated to be insured in the UK in 2024,^72^ and some insurance policies may not provide cover for dental illness, or have low financial limits, so cost may still be a problem even for insured rabbits. Only 3.7% of cases in the current study were reported to have been referred for advanced clinical management, which may also reflect financial difficulties and lack of insurance coverage. However, it may also suggest many primary‐care veterinarians feel confident in the appropriate diagnosis and treatment of this commonly seen disease.

The low use of diagnostic imaging and examination under sedation or general anaesthesia could also indicate some welfare improvement opportunities from increased veterinary education regarding options for dental disease diagnosis. In a 2019 survey of UK veterinarians, both general knowledge of rabbit health and disease and confidence in diagnosing, treating and anaesthetising rabbits were reported as significantly lower than for cats and dogs, while frequently seeing rabbits in practice was reported as significantly improving veterinarians' knowledge and confidence.^2^ Rabbits were reported to comprise just 2.0% of presenting animals at UK veterinary practices in 2017, compared to dogs at 64.8% and cats at 30.3%,^73^ so UK veterinarians' confidence in accurately diagnosing and treating rabbits with dental disease may often be low. Veterinary professionals aiming to conduct thorough dental examinations should consider including diagnostic imaging where possible for initial diagnosis, particularly in more challenging cases showing clinical signs typically related to dental disease but with no evident dental abnormalities, to aid in timely diagnosis and initiation of treatment to prevent unnecessary suffering. It is worth noting that the data in the current study only included the use of diagnostic imaging within 7 days of the initial diagnosis of dental disease, but some cases may have undergone diagnostic imaging after this period for the staging of dental disease and treatment planning. A more detailed study is warranted to better understand the clinical rationales for both undertaking and not undertaking diagnostic imaging in rabbits with dental disease.

### Treatments and veterinary recommendations

Tooth trimming using any method was recorded in more than one‐third (34.0%) of dental disease cases in the current study. Tooth trimming included the use of nail clippers for incisor trimming in some cases, but the current study did not extract specific data on this frequency. While systematic research on the effects of clipping teeth with nail clippers currently seems absent for rabbits, piglets were significantly more likely to experience fractures, haemorrhage, abscessation and necrosis of teeth that had been shortened with clippers instead of a mechanical bur, causing severe pain and behavioural issues.^74^, ^75^, ^76^ Numerous authors recommend against using nail clippers for incisor trimming in rabbits because of reports or experiences of incisor fractures, pulp necrosis and permanently altered incisor growth.^8^, ^9^, ^10^, ^12^, ^16^, ^23^, ^29^, ^30^ It is important to ensure that all primary‐care veterinarians are fully aware of the risks of incisor clipping, and it is strongly recommended to trim rabbit teeth using power tools while the animals are sedated or anaesthetised.^10^, ^12^, ^16^, ^23^, ^24^, ^29^

It was encouraging that the vast majority of rabbit cheek teeth trimming was undertaken under sedation or general anaesthesia. However, a small proportion (6.1%) appeared to have the procedure performed without the recorded use of any sedation or anaesthesia. This finding is unlikely to be an artefact caused by failure to document the use of anaesthesia or sedation because specific medications used should always be entered into the EHRs due to high clinical relevance and to facilitate charging clients for the medication. A lack of familiarity regarding treating rabbits that was self‐reported by some veterinarians^2^, ^3^, ^4^ could mean that some of these conscious trimming events may have occurred due to a lack of awareness of the recommended treatment methods, or due to owner or veterinarian concerns about increased anaesthetic risk in rabbits.^34^ Trimming the cheek teeth of a conscious rabbit is likely to cause intense stress and to compromise animal welfare by increasing the risk of iatrogenic injury and associated pain if the rabbit suddenly moves. It is also likely to provide ineffective treatment due to suboptimal visualisation of the oral cavity.^10^, ^12^, ^16^, ^23^, ^24^, ^28^ Therefore, conscious cheek tooth trimming is not recommended.

Dietary modification is widely recommended for rabbits with early dental disease to slow disease progression. This typically includes the provision of more fibrous and abrasive foods with sufficient calcium and vitamin D concentrations, such as leafy green vegetables, grasses or hay.^8^, ^13^, ^14^, ^16^, ^22^, ^23^, ^24^ Following these recommendations may, therefore, help to reduce pain associated with dental disease and reduce the occurrence of previously described gastrointestinal co‐morbidities. A recommendation for dietary modification was recorded in the EHRs of just 21.5% of cases in the current study, although the actual value could be substantially higher if the verbal recommendation was not recorded in the EHR. In addition, for the current study, a recommendation for dietary modification in relation to dental disease was only extracted if this advice was reported in the EHR within 7 days of dental disease diagnosis. Furthermore, it may have been clinically inappropriate to recommend dietary modification for some cases where the dental disease was too advanced or the rabbit's diet was already adequate. Nevertheless, the findings of the current study are a reminder of the importance of routinely emphasising the value of the provision of a suitable hay‐rich and muesli‐free diet for rabbits for the prevention and treatment of early dental disease.^5^, ^6^, ^25^, ^26^

### Mortality

The high proportion of dental disease cases that died with dental disease as a primary or contributory cause in this population (51.2%) reflects its severity as a welfare concern. For 16.8% of rabbits with dental disease that died, dental disease was recorded as the primary reason for their death. For the further 34.4% where dental disease was a contributory factor to death, these rabbits could have been experiencing the previously discussed painful gastrointestinal co‐morbidities, increasing their risk of death. The actual proportion of deaths linked to dental disease is likely even higher than reported here because a cause of death was unrecorded in 19.9% of rabbits that died.

Arguments exist as to whether death itself is a welfare concern for animals, but it is generally accepted that euthanasia to prevent suffering is often in an animal's best interests, assuming the act of dying does not cause additional suffering.^77^, ^78^, ^79^, ^80^, ^81^ Consequently, euthanasia may have been the best available course of action for many of these rabbits to prevent ongoing suffering from the debilitating effects of dental disease and any co‐morbidities. The high proportion of rabbits in this study that died as a direct result of their dental disease may therefore reflect high proportional euthanasia to safeguard rabbit welfare. However, in the current study, no distinction was made between rabbits that were euthanased and those that died another way, so the proportion of rabbits euthanased on welfare grounds is unknown and requires further investigation.

### Limitations

The results of this study relied on complete and accurate dental disease diagnosis and documentation within the EHRs, so the true frequencies of clinical signs, diagnostic and treatment methods, and rabbit deaths may be underreported if some EHRs lacked detail. Also, the results of this study may not be generalisable to all companion rabbits in the UK, as an estimated 27% of rabbits were not registered with a veterinary practice at all in a 2024 survey.^72^ Dental disease is often a hidden disease in rabbits that remains unnoticed until severe clinical signs or co‐morbidities are detected,^6^, ^7^, ^26^ so a lack of routine veterinary examinations may mean the clinical signs seen in this study may generalise poorly to all rabbits suffering from dental disease. Likewise, clinical signs were only recorded in this dataset if they were reported in the EHR within 7 days of dental disease diagnosis, so the relation of clinical signs to the later progression of acquired dental disease could not be established. Some of the extracted clinical signs may also not have been related to dental disease itself; however, it is likely that the attending veterinarians generally felt the clinical signs were somewhat linked to dental disease if they were recorded at the same time as disease diagnosis. Finally, the use of analgesia was not recorded in this study, but this would be useful to assess in future work to gauge veterinarians’ perspectives on the extent to which rabbits with dental disease experience pain that warrants analgesia.

## CONCLUSION

Through retrospective analysis of the clinical records of 2219 companion rabbits with dental disease, the common clinical signs associated with the disease, the frequency of death in relation to the disease and the veterinary diagnostic and treatment methods used by primary‐care veterinarians in the UK have been described. Some of these findings have implications for rabbit welfare, such as the frequency of reduced food intake in rabbits diagnosed with the disease and the disease's association with death in over half of the rabbits recorded to have died in this population. Veterinarians should be encouraged to consider the welfare improvement opportunities from increased use of radiography for staging of dental disease and treatment planning. Regarding treatment, clipping of incisors should never be performed, and other methods of tooth height reduction should ideally be performed under sedation or anaesthesia, especially for the cheek teeth; in some cases, wider access to veterinary training in these techniques may be helpful.

## AUTHOR CONTRIBUTIONS

**Dan G. O'Neill** and **Dave C. Brodbelt** were responsible for acquiring the veterinary data used in the study. **Charlotte C. Burn, Dan G. O'Neill, Joanna Hedley** and **Dave C. Brodbelt** assisted with study conceptualisation, funding acquisition, model building, analysis and interpretation. **Maria A. Jackson** extracted the data for the analysis. **Maria A. Jackson** and **Dan G. O'Neill** prepared the analytic datasets. **Maria A. Jackson** carried out the analysis and initial manuscript creation, with significant contributions from all co‐authors. All authors approved the final version to be published and agreed to be accountable for all aspects of the accuracy and integrity of the work.

## CONFLICT OF INTEREST STATEMENT

The authors declare they have no conflicts of interest.

## ETHICS STATEMENT

Ethical approval was granted by the Royal Veterinary College Ethics and Welfare Committee (reference number SR2018‐1652).

## Supporting information



Supporting Information

## Data Availability

The dataset analysed during the current study is available on Figshare at https://doi.org/10.6084/m9.figshare.27800397
