# Supplementary material for: Dental disease in rabbits under UK primary veterinary care: Clinical management and associated welfare impacts
Source: Vet Rec. 2025 Apr 3;197(5):e5326. doi: 10.1002/vetr.5326 (PMC12412282; doi:10.1002/vetr.5326)
Supplement: Supplementary file 1 — Supporting Information [file VETR-197-e5326-s001.pdf]

Supporting Information: Definitions of clinical signs

S1. Clinical signs, presenting signs, and examination findings recorded on the EPR within seven days preceding or following a record meeting of the case definition. Signs are grouped by the body system that would most likely be affected. Signs may or may not have been directly related to dental disease. Definitions were created with adaptations from Meredith (2008), Harcourt-Brown (2009), Capello (2016), and Studdert (2021).

Body system	Sign	Definition
N/A	None/unrecorded	No clinical signs were recorded on the EHR.
Gastrointestinal	Reduced food intake	The rabbit was recorded to be eating less food than usual, or inappetence, hyporexia, anorexia, or another related term was recorded in the EHR.
	Refusing hay	The rabbit was recorded to specifically be leaving hay uneaten when they would usually eat it, or the record stated that the rabbit had never eaten any hay since the owner acquired them.
	Reduced faecal output	The rabbit was recorded to be producing fewer dry, hard faecal pellets than usual, or the faeces was smaller in size than usual.
	Reduced gut sounds	The owner reported hearing fewer gut sounds (borborygmi) than normal, or the attending veterinary surgeon heard fewer gut sounds than would normally be expected in a healthy rabbit during clinical examination.
	Gastrointestinal stasis	Gastrointestinal stasis was specifically reported in the EPR as a confirmed or differential diagnosis. A gastrointestinal obstruction may or may not have been present. May also have been called ileus, gastric hypomotility, gut stasis, GI stasis, RGIS (rabbit gastrointestinal syndrome), or another related term.
	Bloat	Gastric dilation or dilatation with gas (bloat) was specifically reported in the EHR as a confirmed or differential diagnosis. May also have been called tympany.
	Diarrhoea	The rabbit was recorded to be producing faeces with a liquid consistency, or liquid faeces was seen during clinical examination.
	Not eating caecotrophs	The rabbit was recorded to be leaving uneaten caecotrophs in its living environment, or uneaten caecotrophs were identified on clinical examination.
	Weight loss	Weight loss or reduced body condition was identified by the owner, or specifically mentioned

		in the EHR. May also have been called emaciation, reduced muscle condition, or the rabbit was described as thinner than normal.
Orofacial	Hypersalivation	The rabbit was recorded to be producing more saliva than normal, or excessive saliva was seen around or within the mouth during clinical examination, or wetness on the chin or lips was noted during examination indicating recent hypersalivation. May also have been called slobbers, drooling, or another related term.
	Oral bleeding	Any active external escape of blood from anywhere within the mouth or on the lips was reported by the owner or noticed by the veterinary surgeon during clinical examination.
	Oral ulcer	Any inflamed, damaged area, defect, or break on the epithelial surface within the mouth or on the lips was reported by the owner or noticed by the veterinary surgeon during clinical examination.
	Oral abscess	A localised collection of pus within a cavity on the cheeks, within the mouth, in the jaw, or around the eyes (including retrobulbar) was noted in the EHR. May have included periodontal, periapical, or iatrogenic abscesses following tooth removal. Ear base swellings and abscesses were NOT included.
	Other or unspecified oral mucosa change	Any other abnormality of the oral mucosa was noted during clinical examination that does not fit any other oral problem definition. May have included hyperplasia, lacerations, trauma, or another related term.
	Mandibular or maxillary swelling	A swelling or change to the usual smooth feeling of bone on the maxilla and/or mandible was noted by the owner, or during clinical examination.
	Ocular discharge	The rabbit was recorded to have excessive discharge of any type from one or both eyes, or a crusting or damp area below the eye(s) by the owner or during clinical examination. May also have been called hyperlacrimation, epiphora, conjunctivitis, dacryocystitis, or another related term.
	Exophthalmos	The rabbit was recorded to have one or both eyeballs bulging and protruding from the usual position within the socket.
Respiratory	Nasal discharge	The rabbit was recorded to have excessive discharge of any type from one or both nares, or a crusting or damp area below the nares by the owner or during clinical examination. May also

		have been called rhinitis, rhinorrhoea, snot, or another related term.
	Any respiratory changes	The rabbit was recorded to display respiratory signs other than nasal discharge by the owner or during clinical examination. May have been called sneezing, dyspnoea, stridor, stertor, wheezing, respiratory tract infections, or another related term.
Dermatological	Perineal faecal accumulation	The rabbit was recorded to have faeces (usually caecotrophs) built-up and impacted around the perineal area, either identified by the owner or on clinical examination.
	Myiasis	The rabbit was recorded to have flystrike (cutaneous myiasis) on the body, indicated by fly larvae (maggots) usually on the perineal area, either identified by the owner or on clinical examination.
	Overgrooming	The rabbit was reported to be grooming itself more frequently or more intensely than usual by the owner.
	Reduced grooming	The rabbit was reported to be grooming itself less frequently or less intensely than usual by the owner.
	Other or unspecified dermatological change	The rabbit was recorded to have a dermatological change other than those previously described. May have included alopecia, pruritis, dermatitis, mite infestations, pigmentation changes, growths, or other related terms.
Behavioural	Lethargy	The rabbit was described to be sleepier or having less energy than normal by the owner. May also have been called drowsiness, inactivity, sluggishness, or another related term.
	Bruxism change	The rabbit was reported to perform bruxism (grinding of the teeth) more or less frequently than normal by the owner, or the bruxism was described to be louder or quieter than normal.
	Other or unspecified behavioural change	The rabbit was reported to display other behavioural changes that it usually did not perform by the owner. May have included aggression, scratching, withdrawal, avoidance, reduced sociability, abnormal toileting, or another related term.
Systemic	Pale mucous membranes	The mucous membranes were recorded to be pale on clinical examination.
	Prolonged capillary refill time	The capillary refill time was recorded to be prolonged on clinical examination.

	Trauma	The rabbit was recorded to have experienced a physical injury (trauma) anywhere on the body, either identified by the owner or on clinical examination.
	Abnormal temperature	The rabbit was recorded to have an abnormally high or low temperature (pyrexia, hyperthermia, or hypothermia) on clinical examination.
Neurological	Any neurological signs	The rabbit was recorded to display any signs of neurological dysfunction by the owner or on clinical examination. May have included vestibular signs such as head tilt, circling, rolling, nystagmus, ataxia, wobbliness, incoordination, or another related term.
Genitourinary	Any genitourinary signs	The rabbit was recorded to display any signs of genitourinary dysfunction by the owner or on clinical examination. May have included pollakiuria, dysuria, haematuria, urinary incontinence, urine scalding, abnormal discharge, or another related term.
N/A	Dead on arrival	The rabbit was recorded to be presented dead on arrival to the practice.

Abbreviations: CRT, capillary refill time; EHR, electronic health record; MMs, mucous membranes.

References

Capello, V. Diagnostic Imaging of Dental Disease in Pet Rabbits and Rodents. *Veterinary Clinics of North America: Exotic Animal Practice*. 2016;19:757–782

Harcourt-Brown, F. Dental disease in pet rabbits 3. Jaw abscesses. *In Practice*. 2009;31:496–505

Meredith, AL. Gastrointestinal Disease in the Rabbit. Proceedings of the WSAVA/FECAVA World Small Animal Congress; 20-24 August 2008, Dublin: WSAVA, 2008:262–264

Studdert, VP, Gay, CC, Hinchcliff, KW. Saunders Comprehensive Veterinary Dictionary. 5th edn. St Louis: Elsevier, 2021
